# Effects of Disease on the Teeth

**Published:** 1860-08

**Authors:** Abr. Robertson


### EFFECTS OF DISEASE ON THE TEETH.

BY ABR. ROBERTSON, D. D. S., M. D.

In 1852, I read an essay before the American Society of Dental Surgeons, which was published in most, if not all, the dental journals of that time, and was intended to show some of the effects of carious teeth on the general health.

I now propose to consider some of the effects of impaired general health—of sickness—on the teeth, and to correct, as far as I may, what I conceive to be a very erroneous and very general impression in relation to the effects of medicines upon the teeth, when administered for the cure or for the relief of diseases.

But as it is not my province to treat the diseases here alluded to, not being a practitioner of general medicine, I shall be obliged to rely mainly on the recorded observations of others for many facts, but whose testimony may be all the more convincing and reliable from the consideration that their observations have been made and the results recorded simply as scientific physiological facts, without reference to any preconceived theory or argument.

The dentist hears no other expression half so frequently as the following, and he always hears it so nearly in the same language as to make it seem as if stereotyped; he hears it, indeed, almost every day, and from all ranks and grades of society—from the most learned and intelligent to the most ignorant and thoughtless: "My teeth were perfectly sound until I was sick, when I took so much medicine that it destroyed them;" or, "since my health has been bad, I have taken so much medicine that it has ruined my teeth." I have known dentists, too, and some of them eminent in their calling, to tell their patients that their teeth had been ruined by taking medicines, and I believe that physicians even sometimes admit the correctness of the charge!

An expression so universal and so common as this; and the expression, I suppose, is no more common than the belief in its correctness is universal—must have some foundation, and, upon the old maxim that "what everybody says must be true," a very strong foundation. Still, paradoxical as it may seem after this admission, it is usually as wide from the facts as any expression well can be—as diametrically opposed to the truth as the old belief that the sun revolves around the earth.

This, like all other fundamental errors, is productive of great harm. It is an unmerited reproach, an ungrateful stigma, upon that most learned and generous, most brave and benevolent of professions, the medical profession, and their most comprehensive and useful art. It is also a direct injury to those who believe the sentiment. It mars their confidence in their medical advisers, and frequently deters them from, at least willingly, taking the medicines properly prescribed by them for the cure of their disease.

But do not medicines injure, and often destroy the teeth? Very rarely, if ever, when judiciously and properly taken. If medicines so rarely injure the teeth, what is the foundation of this, so universal a belief? To answer this question, and to show that medicines properly administered tend to preserve rather than to destroy the teeth, is the chief object of this monograph.

Teeth, beyond doubt, decay more rapidly and more frequently after the occasions for the taking of the medicines have occurred, than before. Why? I propose to answer this question as fully as my present means will allow, though not so fully in detail as I could wish, for want of the definite and certain knowledge that is necessary, and which, though I have sought somewhat assiduously, I have not been able to obtain. But I think, from my own observations, and such observations of others as I have been able to avail myself of, there is sufficient evidence to prove the correctness of my views by specific facts as well as on general principles.

First, we will examine the question negatively. Teeth are not destroyed by medicines.

Chemistry proves that most articles used as medicines neither have nor can have any direct action on the teeth. The only exception to this rule being the acids, and those few salts whose bases have less affinity for their acids than has the lime of which the teeth are principally composed.

Dr. A. Westcott, of Syracuse, N. Y., made a very extensive and careful series of experiments, which he published in the *American Journal of Dental Science* 1843, with the view of of ascertaining and of showing the effects of almost all articles used either as food or medicines, when brought in contact with the human teeth. They were both interesting and instructive, and worthy the perusal of both professional men and laymen.

His experiments demonstrate most conclusively what has already been stated, that but very few articles prescribed as medicines can have any effect on the teeth, even when brought into and kept in contact with the teeth, to wit: acids, ether, (these are derived from a compound of the acids and alcohol,) and such salts as have bases with less affinity for their acids than has the lime of the teeth.

When these acids are given as medicines, especially when the stronger acids are so given, as the sulphuric, nitric, or muriatic, great care should be used to prevent their coming in contact with the teeth, either by taking them through glass, gum-elastic, or other tubes, or by carefully rinsing the mouth with a solution of carbonate of soda, or some other alkali having a stronger affinity for the acid than has the lime of the teeth, or by both these means; otherwise the teeth may be injured, and, if the taking of the medicine is long continued, they may be destroyed.

In an experience now of more than twenty years of pretty active employment, I have seen, perhaps a dozen—but as I

have not kept a record of them, that may be much too high an estimate—well-marked cases of severe injury to the teeth by the use of acids given as medicines; but the use of these stronger acids is so small a part of the materia medica as to be of comparatively little importance, or at most, to account for but an exceedingly small part of the mischief that is charged to the account of taking medicine.

Of the ethers and salts above named, it is enough to say that they are but little used, and when used, in such small quantities, and their contact with the teeth is slight, and of so short duration, simply passing over them during one act of deglutition, and usually greatly diluted, or most probably, if it is the salts that are being administered, they are either given in the form of pills, or in powders, and covered by some kind of jellies or sauces, so that they do not come in contact with the teeth at all, nor are even appreciated by the taste, instead of the teeth being immersed in a considerable quantity of the medicine, and that of full strength, and there kept for at least forty-eight hours, as they were, in the shortest of Dr. Westcott's experiments, that they can have very little if any influence on the teeth; or, if even all the acid that would come in contact with the teeth during any ordinary course of medication with these substances was entirely neutralized by a single tooth, I doubt whether it would be perceptibly affected by it. Of this, at least, I am quite sure: I have no recollection of ever having seen any well-marked case, or any case, where I had any suspicion that caries of a tooth had been caused by any such remedies, except a very few cases from the free use of the nitrate of silver, and where no precautions had been used to prevent its contact with, or action upon the teeth; and in these cases it has always been used as a local application. But as many teeth, such for example as are natually frail or imperfect in their construction, or have already commenced to decay, cannot bear even slight abrasions or corrosions without endangering their usefulness, and of subjecting their possessors to suffering, rinsing the mouth with a solution of soda, after taking such medicines, or after taking lemon, citric, or other vegetable acids, would be but a prudent precaution.

There is one salt which so pre-eminently has the reputation of producing decay of the teeth more than any, or perhaps than all other medicines, that it seems to require especial notice. It is the submuriate of mercury. That this drug, given to a certain extent, and under certain circumstances, has a specific and very peculiar and deleterious action on the

"surroundings" of the teeth, and that many persons, through its agency—by its attacking and destroying the alveoli in its own peculiar, mysterious and unknown way, or by a secondary action through the system—lose their teeth, is too lamentably true; but it never causes decay of the teeth, or if it does, I have yet to see the first indication of it.

Dr. Westcott, in his experiments, found that teeth placed in a mixture of calomel and water of about the consistency of cream, and allowed to remain there for four months, came out as bright and as clean as when they were put in. And some years ago I placed one tooth, thoroughly cleansed from all foreign matter, into a vial with fifty grains of calomel mixed with about two or three fluid drachms of saliva, and at the end of six weeks no change was perceptible even by the aid of a powerful magnifying glass.

In judging of the effects likely to be produced on the teeth by the taking of medicines, any one who has read or may read the record of Dr. Westcott's experiments, should bear in mind that it would require a great many and very large doses of medicine, and the patient to be a long time in swallowing each dose, to equal one of the baths to which he subjected the teeth on which he experimented. And this other fact should also be remembered. His teeth were in a somewhat different condition from those in the mouth of a patient. They had been removed from their natural connections, and thereby deprived of whatever resistance their little vitality might have possessed. They had also, probably, been carefully cleansed from all extraneous matters before subjecting them to the tests; while teeth in the mouth have a low degree of vitality, affording them some protection, and they are also almost always more or less protected by the fatty and albuminous matter, little though it may be, which is deposited from the saliva, and which is left upon them in the mastication of food, etc., from the immediate contact of acid thus rapidly passed over them. This protection, though comparatively very small, in a single act of deglutition might be sufficient to afford them considerable, if not ample protection. *Dental Cosmos, for June*, 1860.

(To be continued.)

---

Nœvus Cured by Creasote.—Dr. Buzalsky reports in the *Med. Zeit.* the entire removal of a nœvus on a child's temple, by pencilling twice a day with creasote.

☞ Mr. S. H. Harding reports, in the *Lancet*, (April 21,) a case of accidental swallowing of artificial teeth, which, fortunately, passed through the alimentary canal without serious inconvenience to the patient. The following are the details of the case: Mrs. ——— swallowed a gold plate on which were three or four teeth, fixed with clasps at each end of the plate, for attachment to the adjoining teeth. One of the clasps had become bent, and formed a sharp point. Some hours afterwards she complained of severe pain in the stomach which seemed very much distended. Dr. Julius made her lie on her right side, and keep quiet. After the lapse of some hours, the pain suddenly left her, and her expression was, that "it went off with a jerk and a pop." She felt nothing more until about twenty-four hours afterwards, when she suffered severe pain in the caput coli, which persisted for some hours, and then passed off in a similar manner. The next morning Dr. Julius was hurriedly sent for, and on arriving found her in great agony, with a constant desire to pass urine and relieve the bowels. Upon passing a catheter into the rectum, he distinctly felt a hard metalic substance firmly fixed across the bowel, about two inches above the sphincter. He now introduced a three-bladed speculum and dilated the bowel as much as could be borne, and, with the aid of two pair of forceps, he brought down the sharp end of the plate, and then extracted it, with very slight injury to the mucous membrane. In a few days she was quite well; and, as Dr. Julius observes, after a good washing, used the same teeth again to assist in recruiting her strength after the painful ordeal she had passed through. The teeth swallowed were two central and two lateral incisors, with the clasp extending to the first molar. One of them had become displaced, and she neglected to have it adjusted. The consequence was they became loose, and were suddenly swallowed, clasp and all." In all accidents of this kind, the patient should partake freely of mush and milk, and such other articles of aliment as will form large and consistent stools, in order to sheath the bowels and promote the more easy passage of the foreign body through the intestinal canal.

---

The Hospital for Nervous Diseases.—Dr. J. S. Ramskill has been appointed, in connection with Dr. Brown-Sequard, a Physician to the National Hospital for Paralysed and Epileptic.

